# Editorial: Current development on wearable biosensors towards biomedical applications

**DOI:** 10.3389/fbioe.2023.1264337

**Published:** 2023-08-08

**Authors:** Sheng Zhang

**Affiliations:** Ningbo Innovation Center, Zhejiang University, Ningbo, China

**Keywords:** biosensor, wearable, biomedical, sensor, soft material, health monitoring

## 1 Background of wearable biosensors towards biomedical applications

Wearable biosensors have gained significant attention in recent years due to their potential to revolutionize healthcare and biomedical applications (Windmiller and Wang, 2013; Matzeu et al., 2015; Bandodkar et al., 2016; Liu et al., 2017; Bariya et al., 2018; Kim et al., 2019; Mohan et al., 2020; Zhang et al., 2021a; Zhang et al., 2021b; Yokus and Daniele, 2021; Sempionatto et al., 2022; Zhang et al., 2022; Min et al., 2023). These devices are designed to monitor physiological parameters and collect real-time data in a non-invasive and continuous manner. The development of wearable biosensors has seen remarkable progress, driven by advancements in flexible materials, sensor technology, miniaturization, wireless communication, and data analytics. These biosensors can be integrated into various wearable forms such as smartwatches, patches, wristbands, fashion accessories and clothing, making them convenient and unobtrusive for long-term monitoring of vital signs, activity levels, and disease biomarkers. The utilization of wearable monitoring devices enable in-depth understanding of the dynamic biochemical processes within these biofluids by means of continuous, real-time monitoring of biomarkers associated with the wearer’s health and performance. Such real-time monitoring not only provides information related to health and performance but also enhances the management of chronic diseases, as well as alerts users or medical professionals to unexpected or unforeseen situations.

One of the key areas of development in wearable biosensors is their integration with advanced sensing capabilities (Imani et al., 2016; Rodbard, 2016; Emaminejad et al., 2017; Kim et al., 2018; Bandodkar et al., 2019; Guo et al., 2019; Wang et al., 2019; Wiorek et al., 2020; Zhang et al., 2021c; Liu et al., 2021). Traditional biosensors primarily focused on monitoring parameters like heart rate and steps taken. However, the current development of wearable biosensors aims to expand their functionality to detect a wide range of biomarkers and physiological parameters (Seitz, 1984; Muramatsu et al., 1987; Wilson and Gifford, 2005; Zheng et al., 2005; Clark and Lyons, 2006; Mannoor et al., 2012; Kim et al., 2015; Gao et al., 2016). For example, wearable biosensors now have the capability to measure electrocardiograms (ECGs), blood pressure, blood glucose levels, body temperature, respiratory rate, and even track sleep patterns. These advancements enable early detection of various health conditions, personalized healthcare management, and remote patient monitoring, leading to improved health outcomes and reduced healthcare costs.

Another significant aspect of the current development in wearable biosensors is the integration of artificial intelligence (AI) and machine learning algorithms (Bishop, 2006; Ayodele, 2010; Delgado-Povedano et al., 2016; Kumar and Chong, 2018; Tatarko et al., 2018; Ho et al., 2019; João Monge et al., 2019; Sharma et al., 2019; Zhou et al., 2019). The combination of wearable biosensors with AI allows for the analysis of complex data patterns and the extraction of meaningful insights. By leveraging AI algorithms, wearable biosensors can detect anomalies, predict health events, and provide personalized recommendations for individuals (Cui et al., 2020; Lussier et al., 2020; Moon et al., 2020; Potyrailo et al., 2020; Rodríguez-Pérez and Bajorath, 2020; Zeng et al., 2020; Ballard et al., 2021; Zhang et al., 2021d; Yüzer et al., 2022). For instance, AI-powered biosensors can detect abnormal heart rhythms, identify patterns indicating the onset of a seizure, or predict fluctuations in blood glucose levels. These intelligent systems not only empower individuals to take proactive measures for their health but also enable healthcare professionals to provide timely interventions and personalized treatment plans based on real-time data from wearable biosensors.

Overall, the current development of wearable biosensors is focused on enhancing their sensing capabilities, improving user experience and comfort, and leveraging AI to extract actionable insights from the collected data. These advancements hold great promise for the future of healthcare, facilitating early detection of diseases, remote monitoring, and personalized healthcare management.

## 2 Future prospects and challenges

### 2.1 Future prospects

- Enhanced Disease Management: Wearable biosensors have the potential to revolutionize disease management by providing real-time monitoring and personalized feedback. They can enable early detection of diseases, such as cardiovascular disorders, diabetes, and respiratory conditions, allowing for timely intervention and improved treatment outcomes. Wearable biosensors may also play a crucial role in monitoring chronic conditions and adjusting treatment plans based on individual responses.
- Remote Patient Monitoring: With the advancements in wireless communication and data analytics, wearable biosensors can facilitate remote patient monitoring. This is particularly beneficial for individuals who require continuous monitoring, such as those with chronic illnesses or post-surgical patients. By transmitting data to healthcare providers in real-time, wearable biosensors enable timely interventions, reduce hospital visits, and improve patient convenience and quality of life.
- Personalized Medicine: Wearable biosensors, combined with AI and machine learning algorithms, can pave the way for personalized medicine. By continuously monitoring an individual’s vital signs, activity levels, and other biomarkers, these sensors can provide insights into their unique health status and patterns. This information can be used to tailor treatments, medications, and lifestyle recommendations specifically to an individual’s needs, leading to more effective and efficient healthcare interventions.

### 2.2 Challenges

- Data Privacy and Security: The increasing use of wearable biosensors raises concerns about data privacy and security. As these devices collect sensitive health information, it is crucial to ensure that the data is protected and accessible only to authorized individuals. Developers and healthcare organizations need to implement robust encryption, secure data storage, and strict privacy policies to maintain patient confidentiality and protect against potential data breaches.
- Measurement of more various Biomarkers: More emphasis should be placed on advanced forms of biosensors and improved non-invasive biofluid sampling techniques for more comprehensive monitoring of various biomarkers. The incorporation of multianalyte sensing holds the potential not only for a more comprehensive assessment of physiological conditions, but also facilitates active calibration and correction of responses, resulting in enhanced accuracy during monitoring procedures. Furthermore, greater efforts should also continue to highlight the incorporation of multiple sensing approaches, such as physical, chemical, and electrophysiological for the same analyte, which could lead to improved biosensor reliability.
- Accuracy and Reliability: Wearable biosensors must demonstrate high accuracy and reliability to gain trust in biomedical applications. Sensor calibration, signal processing techniques, and rigorous validation studies are necessary to ensure that the collected data is accurate and consistent. Additionally, factors such as motion artifacts, environmental influences, and individual variations can affect sensor performance, requiring ongoing improvements to enhance the reliability of these devices.
- Regulatory Approval and Standardization: The regulatory landscape for wearable biosensors in healthcare is still evolving. Developers must navigate through complex regulations and obtain appropriate approvals to ensure the safety and efficacy of these devices. Additionally, establishing standardized protocols for data Research Topic, analysis, and interoperability among different devices and platforms is crucial to enable seamless integration and widespread adoption of wearable biosensors in healthcare settings.
- User Acceptance and Adoption: While wearable biosensors hold great potential, user acceptance and adoption remain critical challenges. Factors such as user comfort, design aesthetics, ease of use, and battery life significantly influence user satisfaction and willingness to incorporate these devices into their daily lives. Educating users about the benefits of wearable biosensors and addressing concerns related to privacy, data ownership, and potential health risks are essential for their widespread acceptance and adoption.
- Widespread Commercial Adoption: Wearable biosensors can fulfill the requirement for collecting biomarker data in a noninvasive manner. The utilization of wearable biosensors encompasses a wide range of applications, including but not limited to fitness and recovery, mental health, personalized healthcare, and telemedicine. The significant translational value of these biosensors paves the way for commercialization. After the development of prototypes, thorough testing and verification of design specifications take place. Feasibility for large-scale production using cost-effective manufacturing techniques should be considered. Furthermore, a regulatory compliance strategy is crucial to ensure timely market access for the product. Preclinical investigation plays a vital role in validating product performance, identifying potential failure modes, and refining risk mitigation strategies.

Overcoming these challenges will require collaboration between researchers, healthcare providers, policymakers, and industry stakeholders to develop robust solutions, establish standards, and address ethical and regulatory considerations. It is of great importance to highlight the development of noninvasive biomonitoring applications and their high specificity, speed, portability, low cost, and low power requirements. The ongoing accumulation of substantial data from extensive human studies, coupled with the utilization of efficient techniques for data fusion and mining, would enable early prediction, diagnosis, and timely intervention of diseases. With continued advancements and strategic efforts, wearable biosensors can shape the future of biomedical applications, transforming healthcare delivery and empowering individuals to take control of their health.
